# A Deep Learning Approach to Classify Fabry Cardiomyopathy from Hypertrophic Cardiomyopathy Using Cine Imaging on Cardiac Magnetic Resonance

**DOI:** 10.1155/2024/6114826

**Published:** 2024-04-26

**Authors:** Wei-Wen Chen, Ling Kuo, Yi-Xun Lin, Wen-Chung Yu, Chien-Chao Tseng, Yenn-Jiang Lin, Ching-Chun Huang, Shih-Lin Chang, Jacky Chung-Hao Wu, Chun-Ku Chen, Ching-Yao Weng, Siwa Chan, Wei-Wen Lin, Yu-Cheng Hsieh, Ming-Chih Lin, Yun-Ching Fu, Tsung Chen, Shih-Ann Chen, Henry Horng-Shing Lu

**Affiliations:** ^1^Institute of Computer Science and Engineering, National Yang-Ming University, Hsinchu, Taiwan; ^2^Faculty of Medicine and Institute of Clinical Medicine, National Yang Ming Chiao Tung University, Taipei, Taiwan; ^3^Division of Cardiology, Department of Medicine, Taipei Veterans General Hospital, Taipei, Taiwan; ^4^Department of Biomedical Imaging and Radiological Sciences, National Yang-Ming University, Taipei, Taiwan; ^5^Institute of Statistics, National Yang Ming Chiao Tung University, Hsinchu, Taiwan; ^6^Department of Radiology, Taipei Veterans General Hospital, Taipei, Taiwan; ^7^Department of Radiology, Taichung Veterans General Hospital, Taichung, Taiwan; ^8^Department of Post-Baccalaureate Medicine, National Chung Hsing University, Taichung, Taiwan; ^9^Cardiovascular Center, Taichung Veterans General Hospital, Taichung, Taiwan; ^10^Department of Pediatric Cardiology, Taichung Veterans General Hospital, Taichung, Taiwan; ^11^Children's Medical Center, Taichung Veterans General Hospital, Taichung, Taiwan; ^12^Department of Pediatrics, School of Medicine, National Chung-Hsing University, Taichung, Taiwan; ^13^College of Medicine, National Chung Hsing University, Taichung, Taiwan; ^14^Department of Statistics and Data Science, Cornell University, Ithaca, New York, USA

## Abstract

A challenge in accurately identifying and classifying left ventricular hypertrophy (LVH) is distinguishing it from hypertrophic cardiomyopathy (HCM) and Fabry disease. The reliance on imaging techniques often requires the expertise of multiple specialists, including cardiologists, radiologists, and geneticists. This variability in the interpretation and classification of LVH leads to inconsistent diagnoses. LVH, HCM, and Fabry cardiomyopathy can be differentiated using T1 mapping on cardiac magnetic resonance imaging (MRI). However, differentiation between HCM and Fabry cardiomyopathy using echocardiography or MRI cine images is challenging for cardiologists. Our proposed system named the MRI short-axis view left ventricular hypertrophy classifier (MSLVHC) is a high-accuracy standardized imaging classification model developed using AI and trained on MRI short-axis (SAX) view cine images to distinguish between HCM and Fabry disease. The model achieved impressive performance, with an *F*1-score of 0.846, an accuracy of 0.909, and an AUC of 0.914 when tested on the Taipei Veterans General Hospital (TVGH) dataset. Additionally, a single-blinding study and external testing using data from the Taichung Veterans General Hospital (TCVGH) demonstrated the reliability and effectiveness of the model, achieving an *F*1-score of 0.727, an accuracy of 0.806, and an AUC of 0.918, demonstrating the model's reliability and usefulness. This AI model holds promise as a valuable tool for assisting specialists in diagnosing LVH diseases.

## 1. Introduction

LVH is a common finding in transthoracic echocardiography (TTE) in clinical practice and is often associated with poor cardiovascular outcomes and ventricular arrhythmias [1]. LVH can be caused by arterial hypertension, aortic stenosis, HCM, Fabry disease, or cardiac amyloidosis. However, distinguishing between these different etiologies can be challenging based on morphological features alone. Cardiac magnetic resonance imaging (MRI) can help differentiate LVH caused by different cardiomyopathies through tissue characterization of the myocardium. For instance, native T1 mapping and extracellular volume (ECV) mapping can effectively differentiate Fabry cardiomyopathy from cardiac amyloidosis or HCM, with a decrease in native T1 value in Fabry cardiomyopathy and a significant increase in T1 value and ECV in cardiac amyloidosis [2–4]. Among various cardiomyopathies with LVH, HCM is the most prevalent, with a reported incidence of 1 : 500 (0.2%) [5, 6]. In Taiwan, Fabry disease is particularly interesting due to its incidence of 1/1600 in male newborns, with 82% having a specific gene mutation of IVS4+919G>A [7]. Fabry disease with this mutation is associated with a late-onset cardiac phenotype, with LVH developing in the midfifties [8]. Enzyme replacement therapy is available to treat the deficiency of galactosidase-A enzyme activity in Fabry disease [9]. Nowadays, cardiac MRI is recommended for early identification of Fabry cardiomyopathy from HCM, which is clinically beneficial for timely managements [5, 10]. However, there is limited availability of parametric mapping on cardiac MRI worldwide, and the standard ranges of native T1 values can vary between different MRI scanners [11]. In this study, we aim to use deep learning techniques to differentiate HCM from Fabry cardiomyopathy by analyzing cine images using a universal cardiovascular magnetic resonance sequence in the absence of myocardial tissue characterization. There are challenges associated with training the model, such as variable depth size and frame rate in the data and the need for large GPU memory [12, 13]. Nevertheless, there have been significant advancements in deep learning techniques for medical tasks and domains [13–18], and 3D residual neural networks (3D ResNet) are effective in analyzing 3D medical images, including cardiac MRI [19, 20]. A previous study has used CNN after segmenting SAX cine images to distinguish HCM from other hypertrophic mimics, including cardiac amyloidosis, Fabry disease, and hypertensive hypertrophy [21]. This study will develop the machine learning model to classify HCM from Fabry cardiomyopathy without segmentation of SAX cine images.

### 1.1. Study Population

This study is a retrospective cohort study conducted at a tertiary medical center in Taiwan, enrolling patients with Fabry disease from January 2010 to September 2020 and HCM from September 2016 to March 2019. The identification of Fabry pedigrees was either derived from the newborn screening program in Taiwan, as described previously [7, 22] or diagnosed by the clinical presentation of unexplained LVH. The diagnosis of Fabry disease was readily determined by screening of *α*-Gal A enzyme activity in men or circulating lyso-globotriaosylsphingosine (lysoGb3) levels in women and was subsequently confirmed by the genetic sequencing of the GLA gene [10, 23]. The study cohort included Fabry disease patients with LVH, or those with focal wall thickness ≥ 13 mm. The HCM was diagnosed by distinctive features as reported in the previous literature [24]. Patients with unexplained LVH or focal wall thickness ≥ 15 mm were included in these studies. All HCM patients were excluded from Fabry disease by the survey of enzyme activity in men and lyso-Gb3 in women and cardiac amyloidosis by technetium-99m pyrophosphate scintigraphy imaging or myocardial tissue characteristics on cardiac MRI. LVH is defined as an increase in left ventricular (LV) mass with LV mass index greater than 115 g/m^2^ in men or 95 g/m^2^ in women by transthoracic echocardiography, which is conducted according to the recommendations from the American Society of Echocardiography [25]. The patients with unexplained LVH or focal wall thickness ≥ 13 mm without evidence of pressure overload were performed cardiac MRI in clinical practice. The present study was approved by the Institutional Review Board of Taipei Veterans General Hospital (IRB number: 2020-03-003BC), and a written informed consent was obtained from each patient. The investigation also conformed to the principles outlined in the Declaration of Helsinki.

### 1.2. Cardiac MRI Acquisition Protocol

Cardiac MRI was performed on 1.5 T scanner (GE Optima MR450w, GE Healthcare, Waukesha, Wisconsin, USA) and on 3 T scanner (Discovery MR750, GE Healthcare, Waukesha, Wisconsin, USA). Each study applied a cardiac phased array receiver surface coil and ECG gating. Cine images were obtained by using a steady-state free precession sequence (echo time (TE): 1.2-1.6 ms, repetition time (TR): 3.2-3.6 ms) in a stack of 8 mm thick short-axis slices encompassing the whole ventricles after gadolinium injection and in long-axis slice. On the other hand, patients with device utilized gradient echo sequence to minimize artifact. The late gadolinium enhancement image acquisition occurred 10-15 minutes after intravenous administration of 0.15 mmol/kg and 0.10 mmol/kg gadobutrol (Gadovist, Bayer, Germany) for 1.5 T and 3.0 T scanner, respectively, using an inversion-recovery gradient-echo pulse sequence with individually adjusted inversion time to optimize nulling of normal myocardium (typical TI: 310-380 ms) [26]. Field of view was typically set to 300 mm × 300 mm (may varied depending on patient size), typical voxel size of the images was 1.6 × 2.0 × 8 mm, the TE was 3.1–3.5 ms, and the TR was 6.2–7.6 ms for 1.5 T scanner and TE 2.5-3.1 ms and TR 5.46.6 ms for 3 T scanner. All patients had obtained informed consent for cardiac MRI, and patient with cardiac implantable electronic device was monitored according to standard procedure [27].

### 1.3. Statistical Analysis

We conducted a statistical analysis to demonstrate the baseline characteristics and LV parameters of study cohort. Continuous variables are expressed as the mean ± SD, and categorical variables are expressed as counts with percentages. Independent sample *t*-test and chi-square test were used to compare normally distributed continuous and categorical variables. All statistical analyses were performed using SPSS version 24 (SPSS Inc., Chicago, IL, USA). Statistical significance was set at two-tailed *P* < 0.05. All statistical analyses were performed using SPSS version 24 (SPSS Inc., Chicago, IL, USA). Statistical significance was set at two-tailed *P* < 0.05.

## 2. Materials and Methods

### 2.1. Data Sources

**Figure 1:**
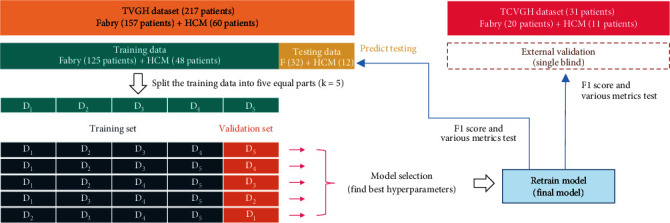
Diagram of internal and external validation processes, including 5-fold cross-validation and model selection.

The research study was conducted under the approval of the Institutional Review Board (IRB) at Taipei Veterans General Hospital (TVGH) and Taichung Veterans General Hospital (TCVGH). The TVGH and TCVGH datasets comprised digital imaging and communications in medicine- (DICOM-) formatted images acquired using GE Healthcare Systems' magnetic resonance imaging (MRI) technology. Specifically, the study focused on the short-axis (SAX) view cine images obtained from MRI scans. We collected 215 MRI images, including 156 images of patients with Fabry disease and 59 images of patients with hypertrophic cardiomyopathies. To evaluate our model's accuracy and generalization ability, we randomly split the data into a training set of 80% and a testing set of 20%. Additionally, we collected 31 MRI images of patients manufactured by Siemens Healthcare Systems from TCVGH to validate our model's generalization ability. All images used in the study had either 512 × 512 or 256 × 256 pixels in size. The TVGH SAX view cine images consisted of 8 mm or 10 mm thick slices with frame rates of 20, 30, or 40. Please refer to Figure 1 for a visual representation of the external verification process.

### 2.2. Implementation Details and Data Preprocessing

In the SAX view, cardiac images contain four dimensions—height, width, depth, and time. Since each patient's heart size and heartbeat vary during the MRI scan, the resulting images have varying slice numbers and frame rates. We adopt several preprocessing steps to standardize the data configurations, including pixel sizes, frame rates, and slice numbers [13]. Firstly, we resize the images to 256 × 256 using bilinear interpolation [28]. Subsequently, we crop the images between 50 and 200 to remove noncardiac organs. To preserve the cardiac cycle, we downsample the sampling rate to a frame rate of 20 using the Fourier method [29].

Next, we select five slices, including the middle and two on either side. While we could consider taking more slices, such as three on each side, this approach could capture unwanted areas, such as the atrium, or miss the heart, leading to a higher computational burden and lower information gain. Finally, we stack the time and depth dimensions together, resulting in a video with a shape of 100 × 150 × 150. We apply min-max normalization to the resulting video to rescale the values between 0 and 1 [30].

### 2.3. Designed Models

For this study, we divided the dataset into 80% for training and 20% for testing. To select the best hyperparameters for our model, we employed stratified 5-fold cross-validation [31]. We evaluated the *F*1-score metric to identify the optimal mean validation *F*1-score and epoch. Subsequently, we trained a final model using 80% of the training dataset and the identified optimal hyperparameters. Additionally, we calculated various metrics based on the mean and standard deviation of the 5-fold cross-validation results [21].

### 2.4. Data Processing Flow

**Figure 2:**
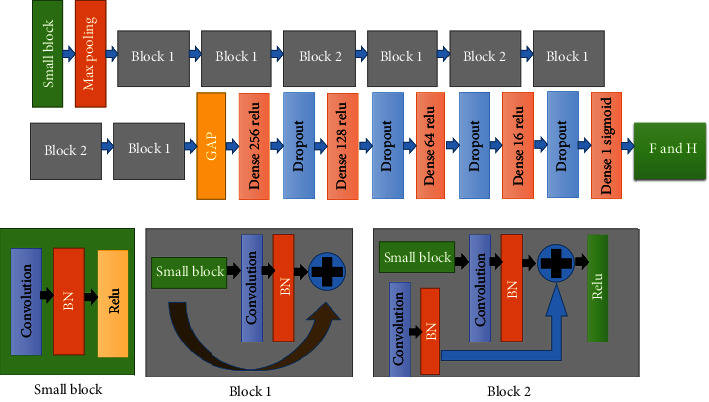
Model architecture based on 3D CNN. Block 1 and block 2 typically refer to different parts of the network designed to process features at different levels. Block 1 usually refers to the initial few layers of the network, primarily responsible for extracting low-level features such as edges and colors. Block 2 represents the subsequent stage in the network, tasked with extracting higher-level features compared to block 1. The stacking of these layers enables the network to learn more abstract and complex features, such as textures and shapes.

**Figure 3:**
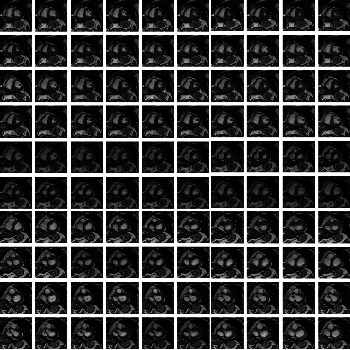
Depiction of selected frames from a 3D MRI sequence viewed in the short-axis orientation to demonstrate the application of augmentation technique through random rotation.

This model is aimed at identifying the direction of a video shot in MRI. Depending on the scanning process, video files may be obtained in either a ventricle-to-atrium or an atrium-to-ventricle direction, which can affect the development of the model. To address this issue, we developed a model that automatically identifies the order in which the images were collected, thus enabling us to standardize the order from ventricle to atrium. We utilized a 3D convolution neural network (3D CNN) structure, specifically the 3D ResNet18 model, as shown in Figure 2 [20]. Since we needed to perform two-class classification and the output of 3D ResNet18 is in 2048 dimensions, we connected four dense layers and dropout layers after each dense layer to avoid bottlenecks caused by extreme compression [32]. To prevent overfitting, we also incorporated dropout layers, L2 regularization, label smoothing, and augmentation techniques, such as random rotation from −*π*/6 to *π*/6, which effectively doubled our dataset by creating a reverse order. It is important to note that we rotated the video (i.e., all images must rotate at the same angle), as illustrated in Figure 3. We used class weight [33] to address the imbalance problem. Specifically, the TVGH image dataset had 153 instances of atrium-to-ventricle and 63 instances of ventricle-to-atrium. In comparison, the TCVGH image dataset had 13 instances of atrium-to-ventricle and 18 instances of ventricle-to-atrium. We used 5-fold cross-validation to identify the optimal hyperparameters and fine-tune the model. Ultimately, this model can differentiate the order of shot images and standardize the order of videos. It is worth noting that all the algorithms for LVH disease classification were implemented using the TensorFlow 2.10.0 deep learning framework on a desktop computer running the Linux Ubuntu operating system. The TensorFlow platform was deployed on a system equipped with an Intel i7-8700K processor and 62 GB of memory and an NVIDIA GTX-1080 Ti GPU boasting 12 GB of memory.

### 2.5. LVH Disease Classification Model

The model is aimed at distinguishing between Fabry disease and HCM disease. The model structure in Figure 2 is the same as the identity order model, except that the fully connected (FC) layer has some differences and does not perform reverse order augmentation. After standardizing the imaging direction for all images (from the ventricle to the atrium), we train the model. There are two challenges with this dataset.

The first is an imbalance problem, where Fabry disease has 156 patients and HCM has 59 patients. To address this issue, we use class weights calculated based on the samples in each class in the training set [33].

The second challenge is overfitting, a common issue in deep learning with small datasets. To avoid overfitting, we add dropout layers after dense layers. Additionally, we add norm two penalties to the ResNet18 and the fully connected layer. The dropout rate is set to 0.4, the fraction of input units to drop. The CNN's L2 regularization factor (*λ*) is 0.00003, while the L2 regularization factor (*λ*) of the fully connected layer is 0.005. We set the label smoothing to 0.1, and our loss function is focal loss (where *γ* is 3) [34]. These methods help to focus on difficult-to-distinguish samples.

Furthermore, we use a learning rate of 0.00005, a batch size of 8, and the Adam optimizer. We train the model for 83 epochs, and these parameters are tuned using 5-fold cross-validation.

## 3. Experimental Results

### 3.1. Baseline Characteristics and LV Parameters

The baseline characteristics and LV parameters measured by echocardiography and MRI are shown in Table 1. In brief, in patients with LVH, there were no significant differences in age, body weight, body surface area, and LV mass index either measured by echocardiography or cardiac MRI in patients with Fabry disease and HCM. However, patients with HCM had thicker interventricular septal wall thickness (IVST) measured by echocardiography compared with patients with Fabry disease. In patients without LVH but only focal wall thickness ≥ 13 mm, patients with Fabry disease were younger, male predominant, thinner IVST, and LV mass index either measured by echocardiography or cardiac MRI.

### 3.2. Results for Identifying Order Models

We evaluated the model's performance using 5-fold cross-validation, testing data, and TCVGH externally validated data during the experiments. The classification results in Table 2 demonstrate high accuracy, providing confidence that the model effectively combines SAX view cine images from the ventricle to the atrium when preprocessed.

### 3.3. Analyze Unified Direction versus Nonunified Direction Training Models

After analyzing Table 3, it was observed that unifying the video direction led to better performance than when it was nonunified. This performance improvement was observed in the 5-fold cross-validation and the TVGH testing set. Consequently, based on this result, the decision was made to unify the training video direction.

### 3.4. Differentiate between Fabry Cardiomyopathy and HCM

In Table 4, we present the performance analysis of our experiment for the classification of Fabry cardiomyopathy and HCM. The evaluation was performed using 5-fold cross-validation, internal testing at TVGH, and external validation at TCVGH. We employed a 5-fold cross-validation technique to evaluate the performance of our model and identify the optimal hyperparameters based on the highest *F*1-score obtained. The best *F*1-score achieved was 0.861, and we utilized this model for training our final classifier for predicting the testing set. Our final model achieved an *F*1-score of 0.861, an accuracy of 0.909, and an AUC of 0.914. However, when it comes to external verification, we face significant challenges due to variations in the equipment, personnel, and practices employed by manufacturers, doctors, and technicians. In addition, our test was a single-blinded study, and we only had one opportunity to demonstrate that our model's performance was acceptable. For the external verification, our model achieved an *F*1-score of 0.727, an accuracy of 0.806, and an AUC of 0.918 when tested on data from TCVGH. Despite the challenges of external validation, our model's performance is promising and merits further investigation.

Furthermore, a blinded reader from TCVGH (SW. Chan) interpreted 44 cardiac MRI SAX cine images, consisting of 32 patients with Fabry cardiomyopathy and 12 patients with HCM. The overall diagnostic accuracy was 54.5%. For patients with Fabry cardiomyopathy and HCM, the diagnostic accuracy was 37.5% and 66.7%, respectively. SAX cine images on cardiac MRI can only provide wall motion and morphologic features without tissue characteristics of myocardium, which makes the differentiation between Fabry cardiomyopathy from HCM challenging for human reader.

### 3.5. Model Visualization

**Figure 4:**
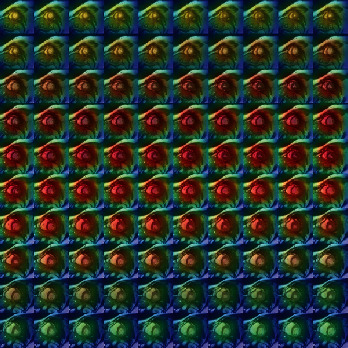
Grad-CAM visualizations illustrating the left ventricular hypertrophy. These images show that the model focuses on cardiac features without the problem of shortcut learning.

Grad-CAM (gradient-weighted class activation mapping) [35] is a CNN visualization technique that enables the identification of model features and the avoidance of shortcuts to determine the model's accuracy. By presenting slices and frames from both cases, Grad-CAM highlights the regions where the model pays the most attention, which helps infer the model's reliability. As shown in Figure 4, the model is focused only on the middle and the heart, with the red portions representing the areas of most significant attention. As a result, the model does not take shortcuts when identifying relevant features.

## 4. Discussions

MRI is frequently used for diagnosing various diseases. However, it is often difficult for clinicians to determine the specific type of ventricular hypertrophy solely based on MRI images. A comprehensive assessment involving medical history, electrocardiogram (ECG), echocardiography, cardiac MRI, and cardiac stress testing is usually required to classify the ventricular hypertrophy type accurately. This process consumes healthcare resources and time. It would be exciting news if we could achieve the same level of accuracy using only one source of imaging data through the robust feature engineering and learning abilities of artificial intelligence (AI). A prior study [36–38] compared our medical imaging research findings for cardiac diseases. Madani et al. [36] primarily focused on the diagnosis of cardiac diseases using echocardiography images, while Zhou et al. [37] and Germain et al. [38] concentrated on the diagnosis of HCM and cardiac amyloidosis using cardiac cine images.

In contrast, our novel approach (MSLVHC) introduces a distinct method for classifying LVH specific to Fabry cardiomyopathy from HCM, utilizing MRI short-axis (SAX) view cine images. In recent years, medical imaging has made significant progress, with magnetic resonance imaging (MRI) being one of the most widely used techniques for diagnosing and monitoring various diseases. As the complexity and quantity of MRI data continue to grow, there is an increasing need for efficient and user-friendly tools to assist healthcare professionals in analyzing and interpreting these images.

### 4.1. Novel Deep Learning Approaches for Cardiac Disease Diagnosis

In this section, we compare the findings of our study with the referenced literature to provide the results and contributions of our research. The studies referenced cover a range of topics in medical imaging, including cardiac disease diagnosis, HCM mutation prediction, and cardiac amyloidosis diagnosis. The four kinds of research all present novel approaches to diagnosing cardiac diseases using deep learning. Madani et al. [36] focus on the diagnosis of cardiac disease using echocardiography images, while Zhou et al. [37] and Germain et al. [38] focus on the diagnosis of HCM and cardiac amyloidosis, respectively, using cardiac cine images. Our approach (MSLVHC) presents a new method to classify LVH for Fabry cardiomyopathy from HCM using MRI short-axis (SAX) view cine images.  Table 5 compares the four approaches based on data sources, dataset, technology (AI method), model design, results, advantages, and disadvantages.

The effective handling of 3D cardiac MRI data is a crucial aspect in advancing the field of medical imaging, and each of the discussed papers provides unique insights into the application of deep learning for cardiac analysis. In comparing these approaches, it is essential to highlight the strengths and weaknesses of each method and the possible implications for future research and clinical application.

Firstly, Madani et al. [36] address the limited labeled medical imaging data challenge through data-efficient deep learning models, specifically semisupervised GANs. The strengths lie in their data efficiency, segmentation for improved classification, and a comprehensive comparative analysis of model architectures. However, the limitations of small sample size and challenges with transfer learning underscore the need for further exploration and validation on larger, more diverse datasets.

Zhou et al. [37] introduce an innovative approach for predicting HCM genetic mutations using deep learning algorithms on cardiovascular magnetic resonance data. Their strengths include a reasonably sized and diverse dataset, comparison with established scores, and enhanced predictive performance when combined with clinical scores. Nevertheless, limitations such as a limited dataset and single-center data raise concerns about the model's generalizability and broader utility in cardiac analysis.

In contrast, Germain et al. [38] propose a novel application of deep learning for diagnosing cardiac amyloidosis using a CNN on cine-CMR images. Their strengths lie in an innovative methodology, an analysis based on patients, and a comprehensive explanation of their methods. However, the limited explainability of CNN decisions, the monocentric nature of the study, and a relatively small sample size suggest areas for improvement and further validation.

When assessing the effective handling of 3D cardiac MRI data, employing 3D CNN, such as the 3D ResNet18, is considered advantageous. This is attributed to their ability to analyze spatial and temporal information in cardiac MRI sequences thoroughly. On the other hand, the disadvantages of the proposed method focus on the limitation in generalization to diverse datasets. This aligns with the weaknesses identified by Madani et al. and Zhou et al., where small sample sizes and single-center data raise concerns about the broader applicability of their models. The proposed mitigation plan, involving collecting additional data from diverse regions and testing on datasets from multiple sources, is crucial for addressing this limitation and enhancing the generalizability of the proposed method.

There is a limitation in our study. In our study cohort, we included half of patients without LVH defined by LV mass index measured at papillary muscle level of parasternal short-axis view on transthoracic echocardiography according to the American Society of Echocardiography guideline [25] but only exhibited with focal wall thickness ≥ 13 mm, which is also reported as LVH. This is the clinical scenario that patients with Fabry disease or HCM can exhibit similar hypertrophic subtypes as concentric or asymmetric ventricular hypertrophy. In this subgroup, patients with HCM had thicker IVST and LV mass index compared with those with Fabry disease, which might constitute a bias for classification and assist our AI model to yield the high accuracy in differentiating ventricular hypertrophy resulted by HCM or Fabry disease. However, this is the real-world practice for exploring patients with unexplained ventricular hypertrophy, and our AI algorithm works for variable phenotypes of hypertrophic form in patients with both diseases.

Overall, to handle 3D cardiac MRI data effectively requires careful consideration of model architecture, dataset characteristics, and generalization capabilities. Each of the discussed papers contributes valuable insights and approaches to cardiac imaging, and future research should focus on addressing the identified limitations to ensure the robustness and applicability of deep learning models in clinical settings.

## 5. Limitations

There are limitations in this study. First, this is a single-center, retrospective cohort study with limited patient datasets and external validation data. The small study cohort may impact the generalizability of our model. Second, there are various hypertrophic phenotypes in patients with HCM or Fabry cardiomyopathy. Patients who exhibited unexplained focal LV wall thickening, apical hypertrophy, or asymmetrical hypertrophy might not meet the criteria to define LVH from the American Society of Echocardiography guidelines. However, our study cohort presented various real-world types of LV hypertrophy, which makes our model applicable in daily practice. Furthermore, we recognize the potential confounding effect of the distribution of patients among different magnetic resonance scanners. As discussed in the article by Kushol et al. [39], acknowledging the significance of scanner bias is crucial. Even though we have utilized cross-validation to enhance the robustness of our model in hopes of reducing the bias introduced by different scanners, the potential bias cannot be completely eliminated.

## 6. Conclusion

The study introduces an AI-driven MRI analysis approach, MSLVHC, distinguishing between HCM and Fabry disease with impressive internal validation results (*F*1-score 0.846, accuracy 0.909, AUC 0.914). External validation at Taichung Veterans General Hospital showed promising performance (*F*1-score 0.727, accuracy 0.806, AUC 0.918). Challenges include diverse data acquisition, which will be addressed by promoting automated techniques for DICOM files and collaboration with data providers. Ongoing research is aimed at refining models for early detection of LVH disease, potentially improving patient outcomes and healthcare decision-making.

## Figures and Tables

**Table 1:** The baseline characteristics and LV parameters evaluated by echocardiography and MRI.

Variables	LVH (*n* = 113)			No LVH but focal wall thickness ≥ 13 mm (*n* = 102)		
	Fabry (*n* = 73)	HCM (*n* = 40)	*P* value	Fabry (*n* = 83)	HCM (*n* = 19)	*P* value
Age (yrs)	58.6 ± 10.0	61.1 ± 13.2	0.27	49.1 ± 13.3	56.4 ± 14.4	0.04
Male, *n* (%)	41 (56.2)	21 (52.5)	0.84	63 (75.6)	9 (47.4)	0.02
Body weight (kg)	64.6 ± 12.7	67.6 ± 13.5	0.24	67.9 ± 13.1	69.2 ± 11.6	0.7
Body surface area (kg/m^2^)	1.70 ± 0.20	1.74 ± 0.22	0.27	1.76 ± 0.20	1.780.19	0.64
IVST (mm)	14.6 ± 3.81	17.5 ± 4.12	<0.001	10.7 ± 2.81	12.9 ± 3.64	0.008
LV mass index by TTE (g/m^2^)	163.4 ± 68.8	161.1 ± 38.1	0.84	84.3 ± 14.8	96.5 ± 11.7	0.003
LV mass index by MRI (g/m^2^)	80.2 ± 28.7	78.6 ± 19.9	0.57	53.2 ± 13.6	66.8 ± 10.7	<0.001
LVEF by TTE (%)	63.3 ± 9.10	64.3 ± 8.09	0.76	66.1 ± 6.59	66.4 ± 10.6	0.91
LVEF by MRI (%)	54.2 ± 8.28	57.0 ± 7.91	0.08	56.8 ± 5.02	60.4 ± 5.33	0.01

IVST: interventricular septal wall thickness; LV: left ventricular; LVEF: left ventricular ejection fraction; MRI: magnetic resonance imaging; TTE: transthoracic echocardiography.

**Table 2:** The *F*1-score and accuracy results for 5-fold cross-validation, TVGH testing, and TCVGH external validation.

Dataset	*F*1-score	Accuracy
5-fold	1.0	1.0
TVGH	1.0	1.0
TCVGH	1.0	1.0

**Table 3:** Accuracy and *F*1-score were compared between unified and nonunified directions using five-fold cross-validation and TVGH testing.

Dataset	Direction	*F*1-score	Accuracy
5-fold	Unified	0.861	0.924
5-fold	Nonunified	0.857	0.924
TVGH	Unified	0.846	0.909
TVGH	Nonunified	0.769	0.864

**Table 4:** The *F*1-score, accuracy, sensitivity, specificity, and AUC were compared on 5-fold cross-validation, TVGH testing data, and TCVGH external validation data.

Dataset	*F*1-score	Accuracy	Sensitivity	Specificity	AUC
5-fold	0.861	0.924	0.895	0.992	0.967
TVGH	0.846	0.909	0.917	0.906	0.914
TCVGH	0.727	0.806	0.727	0.850	0.918

**Table 5:** Comparison of deep learning approaches for cardiac disease diagnosis.

Feature	Madani et al. [36]	Zhou et al. [37]	Germain et al. [38]	MSLVHC (our approach)
Data sources	Echocardiography images, 2,269 images	Cardiac cine images, 198 images	Cine-CMR images, 241 images	MRI SAX view cine images, 214 images
Patients	Not specified	HCM: 198 (genotype (+): 98, genotype (−): 100)	Cardiac amyloidosis: 119, LVH of other origins: 122	Fabry: 156 HCM: 59
Images	LVH: 462, normal apical 4 chamber (a4c): 1,807	HCM: 198 (genotype (+): 98, genotype (−): 100)	Cardiac amyloidosis: 119, LVH of other origins: 122	Fabry: 156, HCM: 59
AI method	Deep learning	Deep learning	Deep learning	Deep learning
Model design	Generative adversarial networks (GAN)	CNN (InceptionResnetV2)+RNN (LSTM)	CNN (VGG)	3D CNN (3D ResNet18)
Results	*F*1-score (0.83), accuracy (0.92), AUC (-)	*F*1-score (-), accuracy (0.84), AUC (0.84)	*F*1-score (-), accuracy (0.83), AUC (0.9)	*F*1-score (0.85), accuracy (0.91), AUC (0.91)
Strengths	The study proposed data-efficient deep learning for medical imaging, leveraging semisupervised GANs to enhance model performance by utilizing labeled and unlabeled data	The study assessed the deep learning model's performance against established genotype prediction scores (Mayo Clinic I and II, Toronto), ensuring a reliable evaluation	The study demonstrated CNN's performance against experienced human operators in visual analysis, assessing its potential to surpass traditional diagnostic methods	The study used a 3D ResNet18, well suited for the cardiac MRI data's inherent 3D nature, effectively capturing spatial and temporal information
Weaknesses	The study recognizes the constraint of a small sample size, urging future research to enhance generalizability through larger and more diverse datasets	The study excludes patients with HCM phenocopies and poor image quality, which may introduce bias, limiting the model's relevance to a broader population	The study was limited to a single institution and a small dataset of cine-CMR images. The generalizability of the deep learning model to other institutions and patient populations is still being determined	The study acknowledges a limitation in generalization capabilities due to the exclusive use of limited datasets from specific hospitals in Taiwan

HCM: hypertrophic cardiomyopathy; LVH: left ventricular hypertrophy.

## Data Availability

The MRI SAX view cine image dataset that supporting this study is provided by Taipei Veterans General Hospital (TVGH) and Taichung Veterans General Hospital (TCVGH), but there are restrictions on the availability of these data, which were used under license for the current study, and so are not publicly available. This dataset is a restricted-access dataset. To access the files, you must be a credentialed user and sign the data use agreement for the project at Taipei Veterans General Hospital.
